# Systemic Treatment with Erythropoietin Protects the Neurovascular Unit in a Rat Model of Retinal Neurodegeneration

**DOI:** 10.1371/journal.pone.0102013

**Published:** 2014-07-11

**Authors:** Stephanie Busch, Aimo Kannt, Matthias Kolibabka, Andreas Schlotterer, Qian Wang, Jihong Lin, Yuxi Feng, Sigrid Hoffmann, Norbert Gretz, Hans-Peter Hammes

**Affiliations:** 1 5^th^ Medical Department, Medical Faculty Mannheim, University of Heidelberg, Mannheim, Germany; 2 Sanofi Diabetes Research and Translational Medicine, Frankfurt, Germany; 3 Institute of Experimental and Clinical Pharmacology and Toxicology, Medical Faculty Mannheim, University of Heidelberg, Mannheim, Germany; 4 Medical Research Center, Medical Faculty Mannheim, University of Heidelberg, Mannheim, Germany; Queen's University Belfast, United Kingdom

## Abstract

Rats expressing a transgenic polycystic kidney disease (PKD) gene develop photoreceptor degeneration and subsequent vasoregression, as well as activation of retinal microglia and macroglia. To target the whole neuroglialvascular unit, neuro- and vasoprotective Erythropoietin (EPO) was intraperitoneally injected into four –week old male heterozygous PKD rats three times a week at a dose of 256 IU/kg body weight. For comparison EPO-like peptide, lacking unwanted side effects of EPO treatment, was given five times a week at a dose of 10 µg/kg body weight. Matched EPO treated Sprague Dawley and water-injected PKD rats were held as controls. After four weeks of treatment the animals were sacrificed and analysis of the neurovascular morphology, glial cell activity and pAkt localization was performed. The number of endothelial cells and pericytes did not change after treatment with EPO or EPO-like peptide. There was a nonsignificant reduction of migrating pericytes by 23% and 49%, respectively. Formation of acellular capillaries was significantly reduced by 49% (p<0.001) or 40% (p<0.05). EPO-treatment protected against thinning of the central retina by 10% (p<0.05), a composite of an increase of the outer nuclear layer by 12% (p<0.01) and in the outer segments of photoreceptors by 26% (p<0.001). Quantification of cell nuclei revealed no difference. Microglial activity, shown by gene expression of CD74, decreased by 67% (p<0.01) after EPO and 36% (n.s.) after EPO-like peptide treatment. In conclusion, EPO safeguards the neuroglialvascular unit in a model of retinal neurodegeneration and secondary vasoregression. This finding strengthens EPO in its protective capability for the whole neuroglialvascular unit.

## Introduction

Rats expressing a transgenic polycystic kidney disease (PKD) gene develop heavy neurodegeneration of photoreceptors due to ciliopathy [1]. This neurodegeneration starts at the first month and is followed by an activation of glial cells [2], [3]. This involves activation of astrocytes and Müller cells, but also microglia. Microglial activation, shown by CD74 upregulation, takes predominantly place nearby capillaries of the deep vascular layer. Cell bodies of CD74-microglia are in contact with the capillaries or are located between capillaries with ramified processes towards them [4]. At the second month of age PKD rats develop an exponential increase in formation of acellular capillaries. This vasoregression is enhanced in the deep vascular layer in comparison to the superficial vascular layer, indicating an influence of activated microglia to vasoregression. In summary the PKD rat develops a damaged neuroglialvascular unit due to transgenic neurodegeneration.

To attenuate this damage a substance influencing all components of the neuroglialvascular unit is necessary. The glycopeptide Erythropoietin (EPO) has various effects besides its proerythropoietic function [5]–[7]. EPO is neuroprotective, which has been shown in various animal studies e.g. in diabetic retinopathy [8], [9]. EPO also protects vessels by strengthening the integrity of endothelial cells and promoting angiogenesis. This effect is mediated by an increase in proangiogenic factors e.g. fibroblast growth factor-2 and VEGF [10]. Interestingly EPO can also lower pathologically increased VEGF-levels in diabetic animals [8]. Another main actor in the pathogenesis of the PKD model, the microglia, is also influenced by EPO. EPO reduces proinflammatory cytokines like interleukin 6, which can switch the phenotype of microglia from a resting to an active status [11].

Influencing the whole neuroglialvascular unit, EPO is a suitable substance to attenuate the retinal phenotype of PKD rats. But due to unwanted side effects of EPO-receptor (EPO-R) stimulation, like thrombosis or promotion of tumor growth, EPO-treatment may not be possible for all patients [12], [13]. However, the protecting effect of EPO in stress situations is rather mediated by the heterodimer of EPO-R and the β-common receptor, called the tissue-protective receptor (TPR), than the homodimeric EPO-R [14]. An EPO-like peptide, exclusively binding to the TPR and not to the EPO-R, has been developed, missing EPOs' effect on hematocrit but still causing protection of the neurovascular unit [15], [16]. In this study we analyze if EPO or EPO-like peptide can safeguard the neuroglialvascular unit in a model of retinal neurodegeneration and secondary vasoregression. To evaluate the protective effects, four week old male heterozygous PKD rats were treated with EPO or EPO-like peptide for four weeks. After this treatment changes in retinal morphology, neurodegeneration and glial cell activity were quantified and localization of pAkt, related to EPO-R activity, was determined. The study revealed that EPO can safeguard neurons and capillaries in this model of retinal photoreceptor degeneration and subsequent vasoregression.

## Materials and Methods

### Animals

PKD-2-247 (PKD) rats, expressing a truncated human polycystic-2 gene, were generated and genotyped as described previously [1]. The rats were exposed to a 12 hours light and dark cycle and had free access to food and drinking water. The study was approved by the ethics committee Regierungspräsidium Karlsruhe, Germany (approval ID: 35-9185.81/G-219/10).

### Treatment with Erythropoietin

At the age of four weeks EPO-treatment started. Heterozygous male PKD rats were injected intraperitoneally three times a week (Monday, Wednesday, Friday) with a suberythropoietic dose of 256 IU/kg body weight with DynEpo (Shire Pharmaceuticals; Basingstoke, United Kingdom), a variant of the human EPO. EPO-treated SD rats and water-injected SD or PKD rats were held as controls. After four weeks of treatment the animals were anesthetized, sacrificed, their eyes were enucleated and stored at −80°C until further analysis.

### Treatment with EPO-like peptide

To avoid unwanted side effects of EPO, EPO-like peptide, missing those effects by signaling via the heterodimeric tissue protective receptor, was used [15]. This peptide, PyrGlu-Gln-Leu-Glu-Arg-Ala-Leu-Asn-Ser-Ser-OH, mimics the external face of helix B of EPO but has an unrelated primary sequence [15]. Homozygous male PKD rats at the age of four weeks were injected intraperitoneally with EPO-like peptide (Bachem Holding AG; Bubendorf, Switzerland) five days a week (Monday till Friday) intraperitoneally with a dose of 10 µg/kg body weight. After four weeks of treatment the animals were anesthetized, sacrificed, their eyes were enucleated and immediately frozen at −80°C until further analysis. Untreated PKD rats at the appropriate age were used as controls.

### Retinal digestion and quantification of retinal morphology

Retinal digest preparations of treated PKD rats and control rats were made to quantify the changes in retinal morphology upon treatment. Eyes were fixed in 4% formalin for at least 48 h. After fixation, retinae were isolated with the help of a stereomicroscope. Retinae were washed with demineralized water for half an hour. Digestion with 5% pepsin in 0.2% HCl occurred for 90 min at 37°C, followed by digestion with 2.5% trypsin in 0.2 mol/l Tris (pH 7,4) for 15–30 min. Unwanted, residual tissue relicts were eliminated by adding water drops to the preparations. After this process of digestion, only the retinal vasculature system remained at the object slide. After dehydration, the retinal vasculature was stained with periodic acid-Schiff and hematoxylin.

Quantification occurred using an Olympus BX51 microscope (Olympus; Tokio, Japan). Endothelial cells, pericytes and migrating pericytes were quantified at 400 fold magnification in ten randomly chosen areas in the middle of the retinal radius using analysis software (Soft Imaging Systems GmbH, Münster, Germany). Endothelial cells and pericytes had been distinguished by their localization, morphology and staining intensity of their cell nuclei. Migrating pericytes were defined as pericytes with triangle shaped nuclei, migrating from vessels with a basal area smaller than the side area [17]. Cell number was quantified in relation to the retinal area. Segments of acellular capillaries were quantified in ten randomly chosen areas in the middle radius of the retina by using an integration ocular (raw data visible in Data S1).

### PAS staining and quantification of retinal thickness and cell number

Paraffin sections of 3 µm thickness were cut. They were deparaffined at 60°C for 90 min and incubated in xylene two times for 5 min. Rehydration occurred in an ethanol dilution series: 5 min in 100%, then 96%, 80% and 70% ethanol (v/v) for 1 min each. Sections were washed in distilled water for 5 min. Then incubation with 0.5% periodic acid solution for 20 min followed to start the oxidation reaction. Sections were washed again in distilled water and then treated with lukewarm Schiff's reagent. After washing in distilled water the sections were dehydrated using an increasing ethanol concentration series: 1 min each with 70%, 80% and 96%, then 5 min in 100% ethanol. Finally sections were twice incubated in xylene for 5 min each. Sections were covered and dried over night.

Quantification of retinal thickness was performed at 50 fold magnification (Leica DMRBE Mikroskop; Leica; Wetzlar, Germany). Two pictures were necessary to illustrate the whole retina. For manual measurement Leica IM50-Software (Leica; Wetzlar, Germany) was used. At every image the measurements were done at three different areas. Thickness of the retinal layers was quantified in a central area, i.e. near the optic nerve, and a peripheral area. Cell nuclei were counted in the same sections. Images at 200 fold magnification were taken from the ganglion cell layer, the inner nuclear layer and the outer nuclear layer and manual counting of the cell nuclei followed. This quantification was also performed in central and peripheral areas (raw data visible in Data S1).

### RNA Isolation

Retinae from frozen eyes were isolated and lysed in 350 µl RLT-Buffer (RNeasy Mini Kit; Qiagen, Hilden, Germany) by the use of needles in downward sizes from 22 to 30 G. The RNA was isolated as described in the protocol. In short, 350 µl 70% ethanol were added, the mix was transferred to the RNeasy spin column and centrifuged for 15 s at 10.000 rpm. After removal of the discharge, washing steps with RW1-buffer and RPE-solution followed. The washing solutions were removed and the spin column was transferred to a new tube. 30 µl RNase-free water were added and centrifugation for 1 min at 10.000 rmp. The Quality of RNA was evaluated by measuring the absorbance at 280 nm. The RNA was stored at −80°C until further application.

### Quantitative real time PCR

QuantiTectH Reverse Transcription kit (Qiagen, Hilden, Germany) was used for the generation and amplification of cDNA. The mix of cDNA, Master Mix and primers was amplified using the ABI PRISM 7700 Sequence Detection System (Applied Biosystems, Darmstadt, Germany). 50°C for 2 min and 95°C for 10 min started the amplification reaction. Then 40 amplification cycles at 95°C for 15 sec and 60°C for 1 min followed. All primers were purchased from Applied Biosystems: CD74 (Rn 00565062_m1), fibroblast growth factor 2 bFgf (Rn 00570809_m1) and ciliary neurotrophic factor Cntf (Rn 00755092_m1). β-actin (Assay no. Rn 00667869_m1) was used as housekeeping gene and the expression quantified using the 2^−ΔΔCT^ method.

### Immunofluorescence of whole mounts

Eyes were fixed in 4% formalin overnight. Retinae were isolated using a stereomicroscope and washed with PBS three times. The tissue was blocked and permeabilized by treatment with 1% BSA and 0.5% Triton-X for 1 h at room temperature. After further washing steps the incubation with the primary antibodies, CD74 1∶50 (sc-5438; Santa Cruz Biotechnology, Santa Cruz, USA) and Lectin-Tritc (L5264; Sigma Aldrich, St. Louis, USA) occurred, diluted in blocking buffer (1% BSA and 0.5% Triton-X) over night at 4°C. Three times washing with PBS was followed by incubation with the secondary antibody donkey-anti-goat Fitc (R125F; Acris Antibodies, San Diego, USA) diluted in blocking buffer for 1,5 h at room temperature. Final washing steps were performed and retinae were fixed on object slides in 50% glycerol. Confocal laser scanning microscopy was used to take the pictures.

### Immunofluorescence of paraffin sections

Paraffin sections were prepared, deparaffined and rehydrated as described before. After washing in phosphate buffered saline (PBS) two times, the sections were blocked with 1% bovine serum albumin and 0.5% Triton-X in PBS for 1 h at room temperature. Blocking solution was removed, sections were washed with PBS and incubated with the first antibody diluted in PBS at 4°C overnight: GFAP 1∶200 (M0761; Dako AG, Wiesentheid, Germany) and pAkt 1∶25 (9266; Cell Signalling, Cambridge, United Kingdom). The next day the sections were washed with PBS and incubated for 1 h at room temperature with the secondary antibody diluted in PBS: rabbit-anti-mouse FITC 1∶200 (F0261; Dako AG, Wiesentheid, Germany) or swine-anti-rabbit FITC 1∶100 (F0205; Dako AG, Wiesentheid, Germany). The sections were washed with PBS and covered with 50% glycerol. Images were taken with confocal microscopy (Leica TCS SP2; Leica, Wetzlar, Germany).

### Statistics

All values are expressed as mean value and standard deviation. Significance was analyzed using Student's t-test and defined as follows: *<0.5; **<0.01; ***<0.001.

## Results

### EPO and EPO-like peptide reduced acellular capillaries in the PKD model

**Figure 1:**
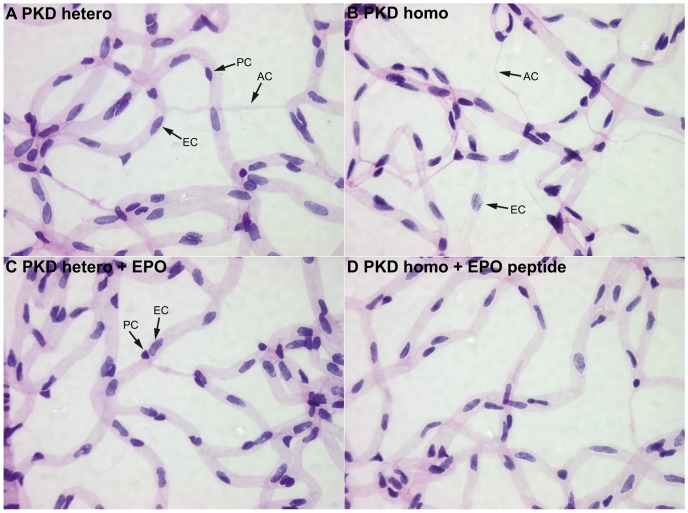
Examples of retinal digest preparations, used to quantify retinal morphology (magnification 400 fold). Endothelial cells and pericytes can be distinguished by morphology, localization and staining intensity. Examples are marked in the pictures. EC endothelial cells, PC pericytes, AC acellular capillaries.

**Figure 2:**
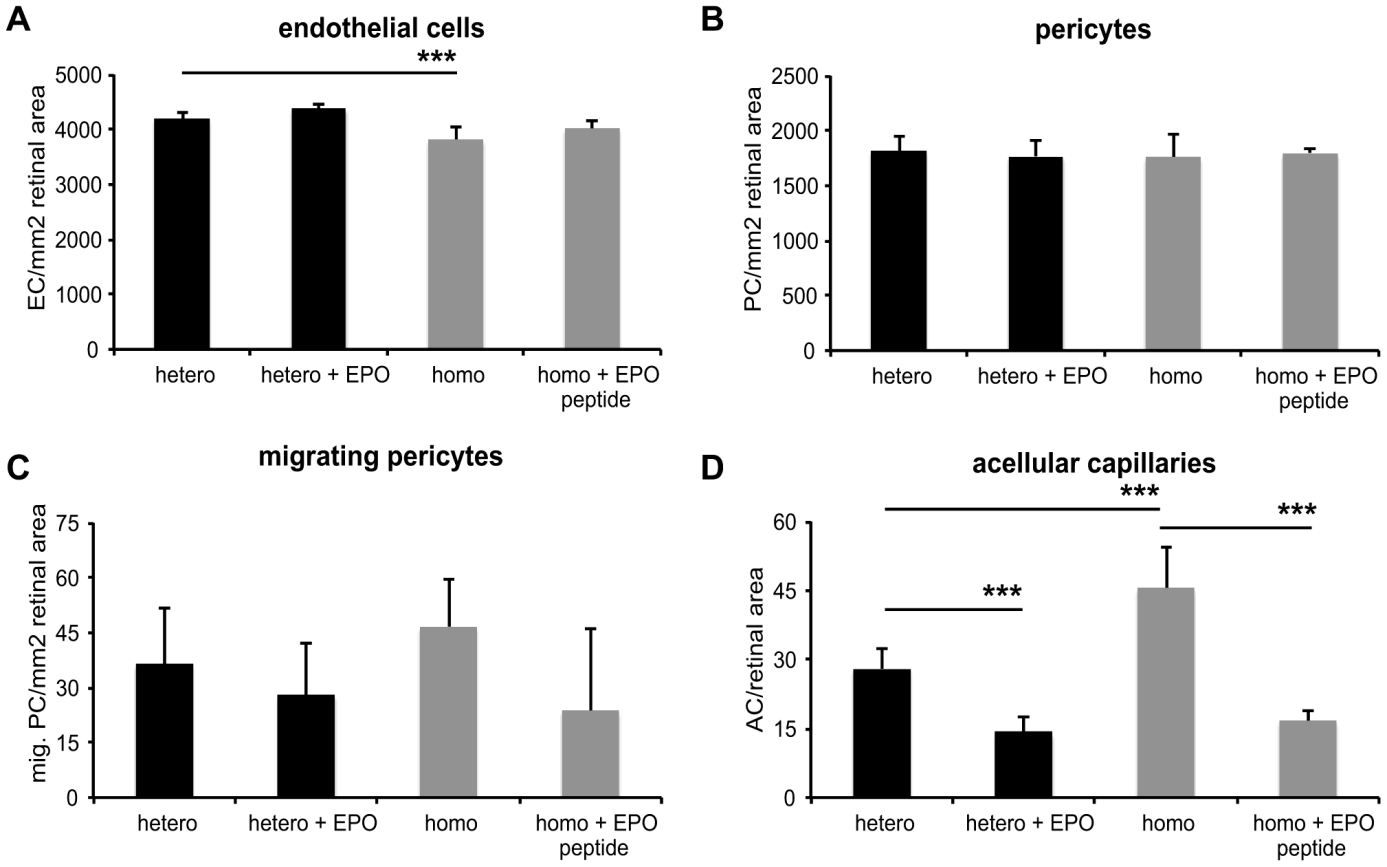
Quantification of retinal morphology after EPO and EPO-like peptide treatment. Four week old heterozygous, male PKD rats were treated with 0.5(n = 6) and homozygous rats with 0.5 ml EPO-like peptide five times a week (n = 4). After four weeks of treatment the animals were sacrificed and their eyes analyzed. Untreated animals (n = 4/5) were held as controls. All values are indicated as mean values ± standard deviation. Significance was evaluated by Student's t-test and defined as follows: *<0.05; **<0.01; ***<0.001. Quantification of endothelial cells (A) showed a decrease by 9% (p<0.001) in homozygous rats compared to heterozygous rats. Treatment with EPO or EPO-like peptide revealed no difference in endothelial cells or pericytes (A, B). Number of migrating pericytes (C) was reduced by 23% or 49% (both not significant). Quantification of acellular capillaries showed a 62% (p<0.001) increase in homozygous compared to heterozygous rats. By EPO treatment, a significant reduction of acellular capillaries by 49% (p<0.001) was achieved. EPO-like peptide reduced the number of acellular capillaries significantly by 63% (p<0.001).

Four week old PKD rats were treated three times per week with 0.5 ml/kg body weight DynEpo (n = 6) or EPO-like peptide (n = 4). After four weeks of treatment the animals were sacrificed, their eyes were enucleated and underwent retinal digestion. Figure 1 shows examples of retinal digest preparations (magnification 400 fold). Quantification of retinal morphology revealed no significant or relevant difference in number of endothelial cells or pericytes between treated and control animals (figure 2, A and B). However, there was a significant reduction in the number of endothelial cells by 9% (p<0.001) in homozygous PKD rats compared to heterozygous PKD rats. The number of migrating pericytes was reduced due to EPO or EPO-like peptide by 23% or 49%, but did not reach statistical significance (figure 2, C). Homozygous PKD rats developed 62% (p<0.001) more acellular capillaries compared to heterozygous PKD rats. EPO-treatment in heterozygous rats reduced acellular capillaries highly significantly by 49% from 28 to 14.17 (p<0.001). Number of acellular capillaries in homozygous rats treated with EPO-like peptide was significantly reduced by 63% from 45.6 to 16.75 (p<0.001) (figure 2, D).

### EPO increased retinal thickness within the range of central photoreceptors

**Figure 3:**
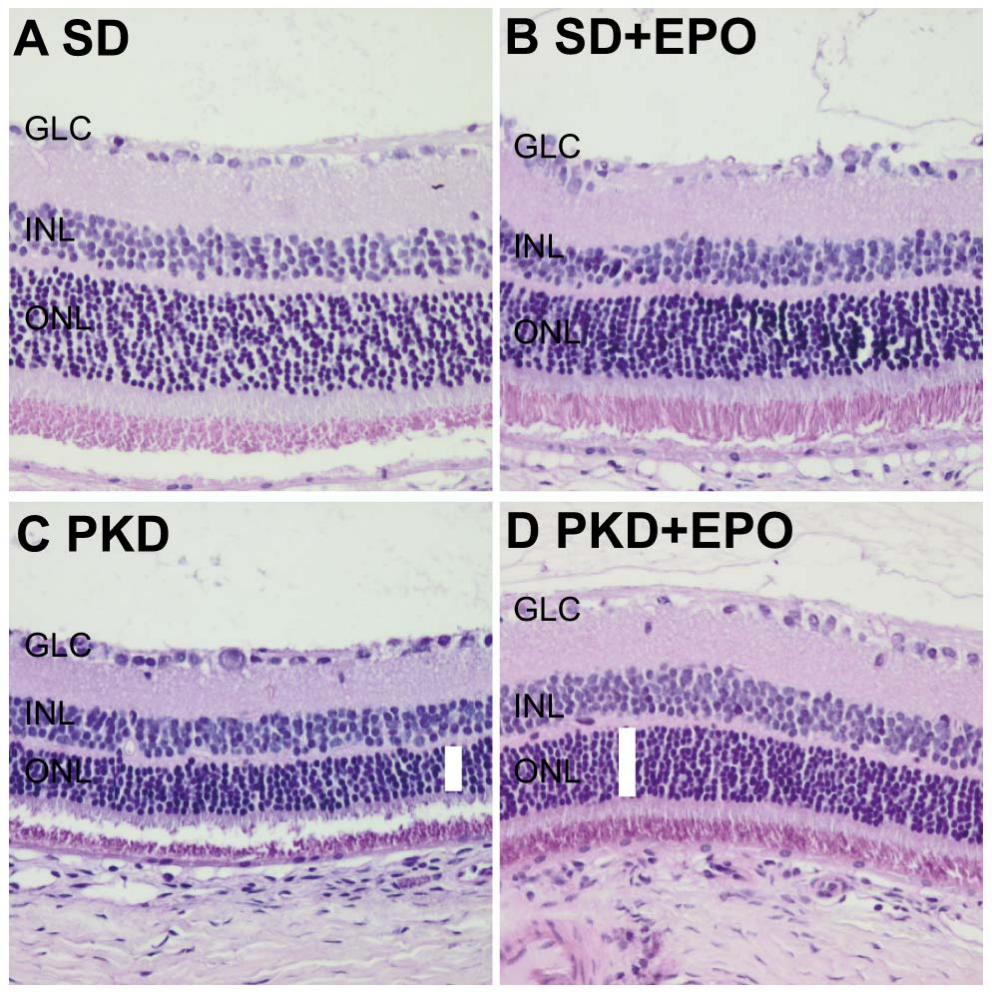
Examples of central retinal sections, used to measure the thickness of retinal layers and count cell nuclei. In these examples 20fold magnification was used for a better overview. Measurement of retial layers was performed using 50fold magnification, cell counting was performed using 200fold magnification. White bars in C and D were used to illustrate the difference in thickness of the outer nuclear layer. GCL ganglion cell layer, INL inner nuclear layer, ONL outer nuclear layer.

**Figure 4:**
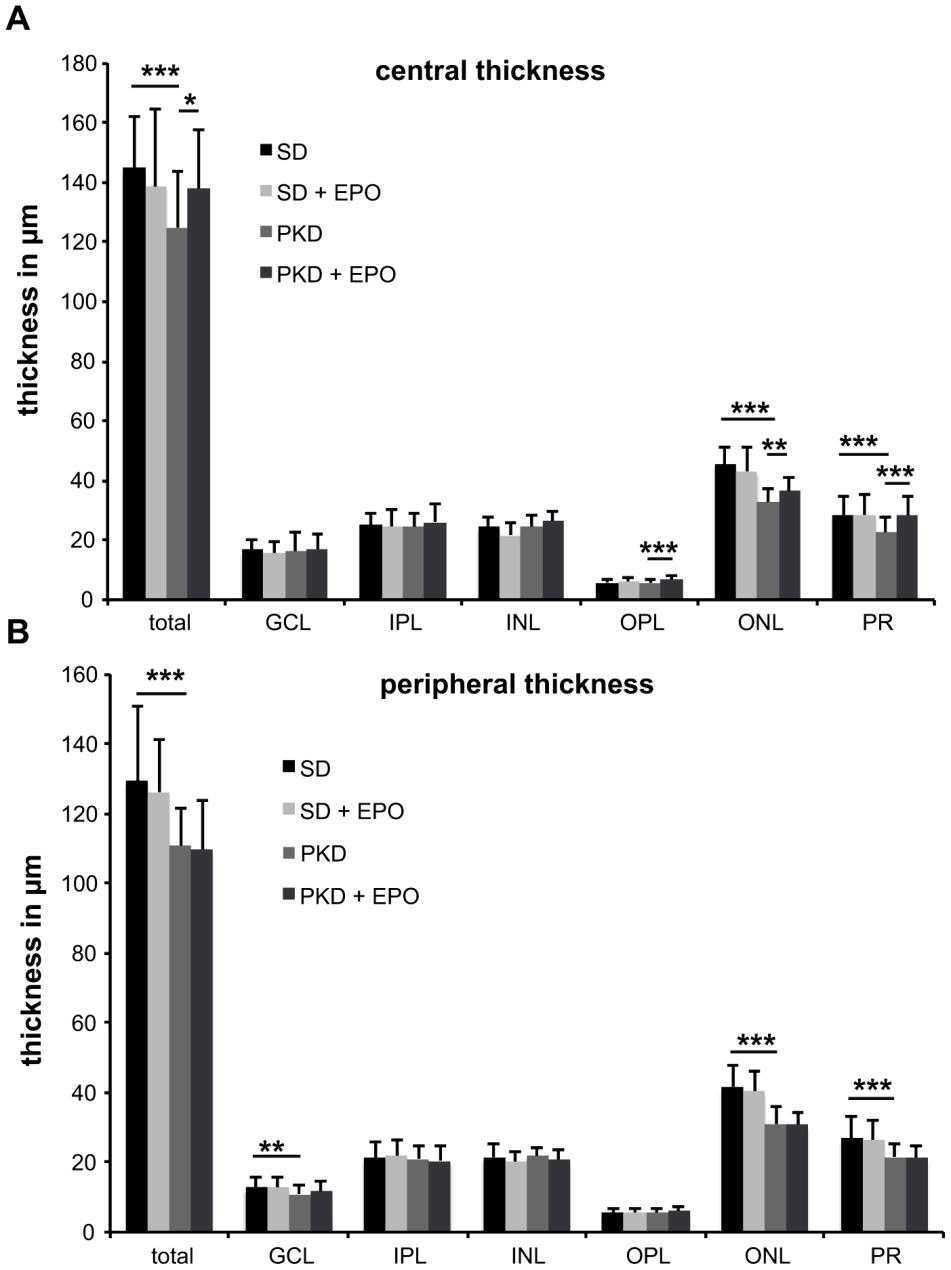
Quantification of central (A) and peripheral (B) retinal thickness after EPO treatment. Four week old heterozygous male PKD rats (n = 4) were treated three times a week with 0.5 ml/kg body weight DynEpo intraperitoneally for four weeks. EPO-treated SD rats (n = 6) and water-treated PKD (n = 5) or SD (n = 6) rats were held as controls. At the age of eight weeks the animals were sacrificed, their eyes enucleated and PAS-stained paraffin sections were prepared. Central, i.e. near the optic nerve, and peripheral thickness were evaluated using a Leica DMRBE microscope and Leica IM50-software. All values are expressed as mean ± standard deviation. Significance was analyzed using Student's t-test and defined as follows: *p<0.05; **p<0.01; ***p<0.001. Central (A) total thickness of PKD retinae compared to SD retinae was reduced by 14% (p<0.001), consisting of a 28% reduction in the outer nuclear layer (p<0.001) and 21% in the outer segments of the photoreceptor layer (p<0.001). EPO treatment increased total retinal thickness in PKD rats in comparison to water-treated PKD rats by 11% (p<0.05), consisting of an increase of the outer plexiforme layer by 19% (p<0.001), the outer nuclear layer by 12% (p<0.01) and the outer segments of photoreceptors by 26% (p<0.001). Total peripheral (B) thickness of PKD retinae compared to SD retinae was reduced by 14% (p<0.001), consisting of a 15% reduction in the ganglion cell, a 26% reduction in the outer nuclear layer (p<0.001) and a 19% reduction in the outer segments of the photoreceptor layer (p<0.001). EPO treatment had no significant effect on retinal thickness in SD or PKD rats. GCL ganglion cell layer, IPL inner plexiforme layer, INL inner nuclear layer, OPL outer plexiforme layer, ONL outer nuclear layer, PR outer segments of photoreceptors

To evaluate the neuroprotective effect of EPO, retinal thickness was measured in SD and PKD rats treated with EPO or vehicle. Figure 3 shows retinal sections (magnification 50fold) used to measure the thickness of the retinal layers. The same sections with a 200fold magnification were used to quantify cell nuclei number. The quantification was done in a central area, i.e. next to the optic nerve, and a peripheral area. In the central area (figure 4, A) the total retinal thickness was reduced by 14% (p<0.001) in PKD compared to SD rats. This total decrease in retinal thickness consisted of a reduction by 28% in the outer nuclear layer (p<0.001) and by 21% in the outer segments of the photoreceptor layer (p<0.001). Through EPO treatment part of this decrease could be prevented. Total retinal thickness increased in EPO-treated PKD rats in comparison to water-treated PKD rats by 11% (p<0.05). This was a composite of an increase in thickness of the outer plexiforme layer by 19% (p<0.001), in the outer nuclear layer by 12% (p<0.01) and in the area of the outer segments by 26% (p<0.001). The peripheral retina (figure 4, B) only showed differences in retinal thickness between SD and PKD rats. Total peripheral retina in PKD rats in comparison to SD rats is reduced by 14% (p<0.001). This reduction consisted of a 15% reduction in the ganglion cell layer (p<0.01), a 26% reduction in the outer nuclear layer (p<0.001) and a 19% reduction in the outer segments of the photoreceptor layer (p<0.001). This data shows that EPO has a neuroprotective effect in the PKD model from which the central photoreceptors benefit.

### No difference in cell number after EPO-treatment

**Figure 5:**
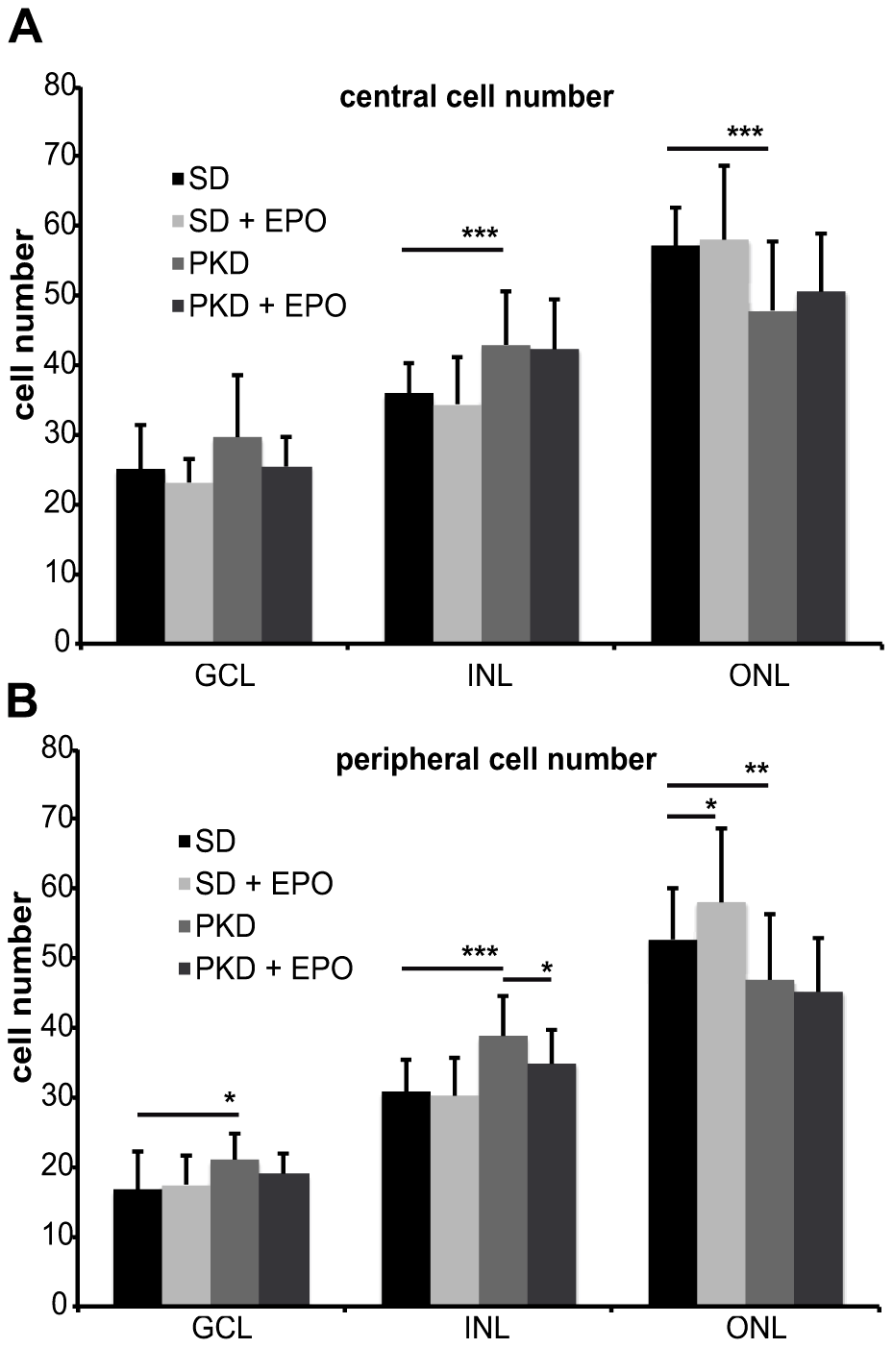
Counting of cell nuclei in SD and PKD rats after EPO-treatment. Four week old heterozygous male PKD rats (n = 4) were treated three times a week with 0.5 ml/kg body weight DynEpo intraperitoneally for four weeks. EPO-treated SD rats (n = 6) and water-treated PKD (n = 5) or SD (n = 6) rats were held as controls. At the age of eight weeks the animals were sacrificed, eyes enucleated and PAS-stained paraffin sections were performed. Cell nuclei were counted in the ganglion cell layer (GCL), the inner nuclear layer (INL) and the outer nuclear layer (ONL) in a central (A) and a peripheral (B) area of the retina. All values are expressed as mean ±standard deviation. Significance was analyzed using Student's t-test and defined as follows: *p<0.05; **p<0.01; ***p<0.001. Central area (A) revealed an increase in the INL by 20% (p<0.001) and a reduction in the ONL by 16% (p<0.001) of PKD rats in comparison to SD rats. EPO-treatment showed no significant effect in the central area of SD or PKD rats. The peripheral areas (B) also revealed significant reduction of 11% in the ONL (p<0.01) and an increase of 25% in the INL (p<0.001) and the GCL (p<0.05) in PKD rats compared to SD controls. EPO-treatment increased the number of cells in the ONL in SD rats by 10% (p<0.05) and decreased the number of cells in the INL of PKD rats by 11% (p<0.05). GCL ganglion cell layer, INL inner nuclear layer, ONL outer nuclear layer

Additional to the measurements of retinal thickness, cell nuclei were counted to evaluate the neuronal status after EPO treatment. As before, counting was performed in a central and a peripheral retinal area. The central retinal area revealed significant differences between SD and PKD rats (figure 5, A). The number of cells in the outer nuclear layer in PKD rats was smaller by 16% (p<0.001), whereas the cell number in the inner nuclear layer was higher by 20% (p<0.001). EPO treatment neither in SD nor in PKD rats showed a relevant or significant change in cell number. Peripheral areas also revealed significant reduction of 11% in the outer nuclear layer (p<0.01), an increase of 25% in the inner nuclear layer (p<0.001) and the ganglion cell layer (p<0.05) in PKD rats compared to SD controls (figure 5, B). EPO-treatment increased the number of cells in the outer nuclear layer in SD rats by 10% (p<0.05). In PKD rats EPO treatment only affected the inner nuclear layer, where it decreased the number of cells by 11% (p<0.05). Together with the previous data this result suggests that EPO rather has an effect on the cell size and/or extracellular matrix than on the absolute number of cells.

### Decreased microglial and Müller cell activity due to EPO-treatment

**Figure 6:**
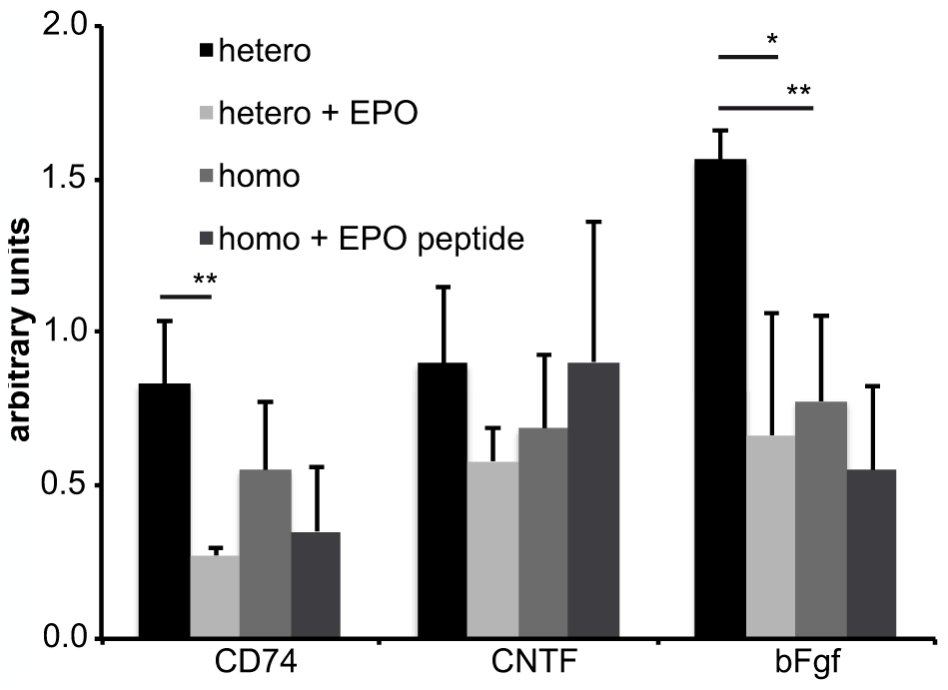
Expression of CD74, CNTF and bFGF after treatment with EPO or EPO-peptide. Four week old heterozygous or homozygous male PKD rats (each n = 5) were treated with 0.5 ml/kg body weight DynEpo three times or EPO-like peptide five days a week intraperitoneally for four weeks. At the age of eight weeks the animals were sacrificed, the eyes enucleated, retinal RNA was isolated and gene expression of CD74, CNTF and bFGF analyzed. All values are expressed as mean ± standard deviation. Significance was analyzed using Student's t-test and defined as follows: *p<0.05; **p<0.01. CD74 shows a reduction by 67% (p<0.01) in heterozygous and by 36% (n.s.). CNTF revealed no significant changes in gene expression. In heterozygous rats it decreased by 35% (n.s.), while it increased in homozygous animals by 32%. bFGF showed a reduction in both groups. It significantly decreased in heterozygous rats by 58% (p<0.01). Homozygous animals revealed 50% (p<0.01) lower expression of bFGF than heterozygous, but still achieved a reduction in gene expression by EPO-peptide of 29% (n.s.).

**Figure 7:**
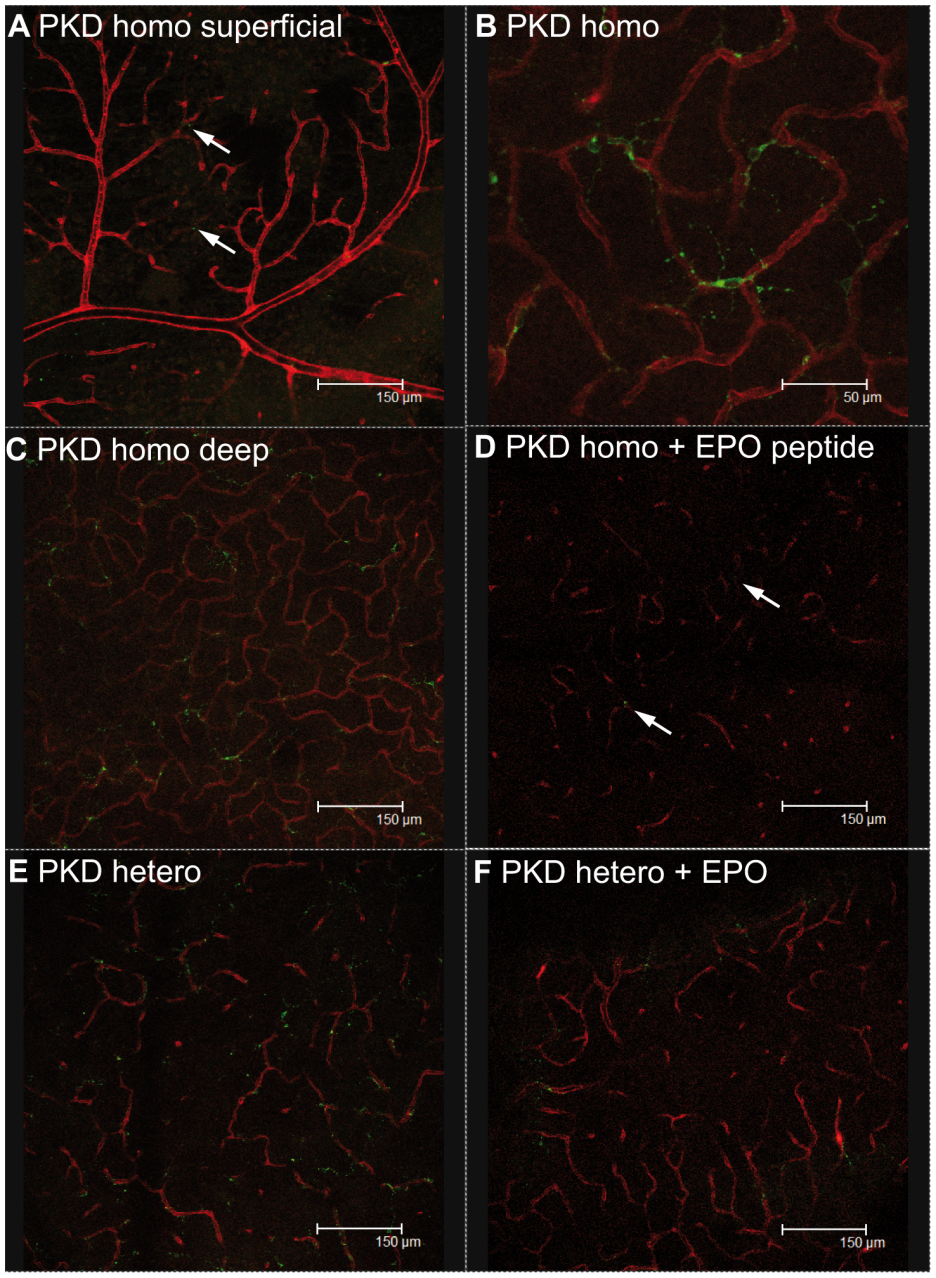
Immunofluorescence staining of CD74 in retinal whole mounts. CD74 is stained with Fitc (green) and Lectin with Tritc (red). A shows a representative example of a superficial capillary layer with almost no CD74 positive cells. B is a magnification of CD74 positive cells with their typical ramified shape. Homozygous PKD rats without and with EPO peptide are shown in C and D. Likewise E and F represent heterozygous PKD retinae without and with EPO treatment. EPO and EPO-like peptide reduce the amount of CD74 positive cells. Arrows mark CD74 positive cells.

The expression of CD74, the most upregulated gene in the PKD model, decreased after the treatment with EPO or EPO-peptide (figure 6). In heterozygous PKD rats the CD74 expression significantly decreased by 67% (p<0.01) upon EPO treatment. In homozygous PKD rats the CD74 expression was one third lower than in heterozygous animals. Still, the EPO-peptide caused a nonsignificant reduction in CD74 by 36% (n.s.). Corresponding results were achieved using immunofluorescence staining of CD74 (figure 7). CD74 positive cells were mainly localized in the deep capillary layer. Heterozygous PKD rats (figure 7, E) exhibited more CD74 positive cells than PKD homozygous rats (figure 7, C). The amount of positive cells was reduced by EPO and EPO peptide (figure 7, D and F). Levels of CNTF and bFGF were evaluated as indicators for the interaction between microglia and Müller cells (figure 6). Changes in CNTF after EPO or EPO-peptide treatment did not reach significant levels. In heterozygous rats EPO reduced CNTF by 35% (n.s.), while EPO-peptide increased CNTF in homozygous animal by 32%. Between homo- and heterozygous animals there was a slight difference in CNTF-expression (24% decrease in homozygous rats; n.s.). The bFGF expression differed clearly due to EPO-treatment. In heterozygous animals a significant reduction of 58% was achieved (p<0.01). bFGF revealed the strongest difference between homo- and heterozygous animals. Homozygous rats had a significantly lower expression of bFGF by 50% (p<0.01). Still it was further reduced by 29% (n.s.) upon EPO-peptide treatment.

### EPO and EPO-like peptide increase the expression of pAkt

**Figure 8:**
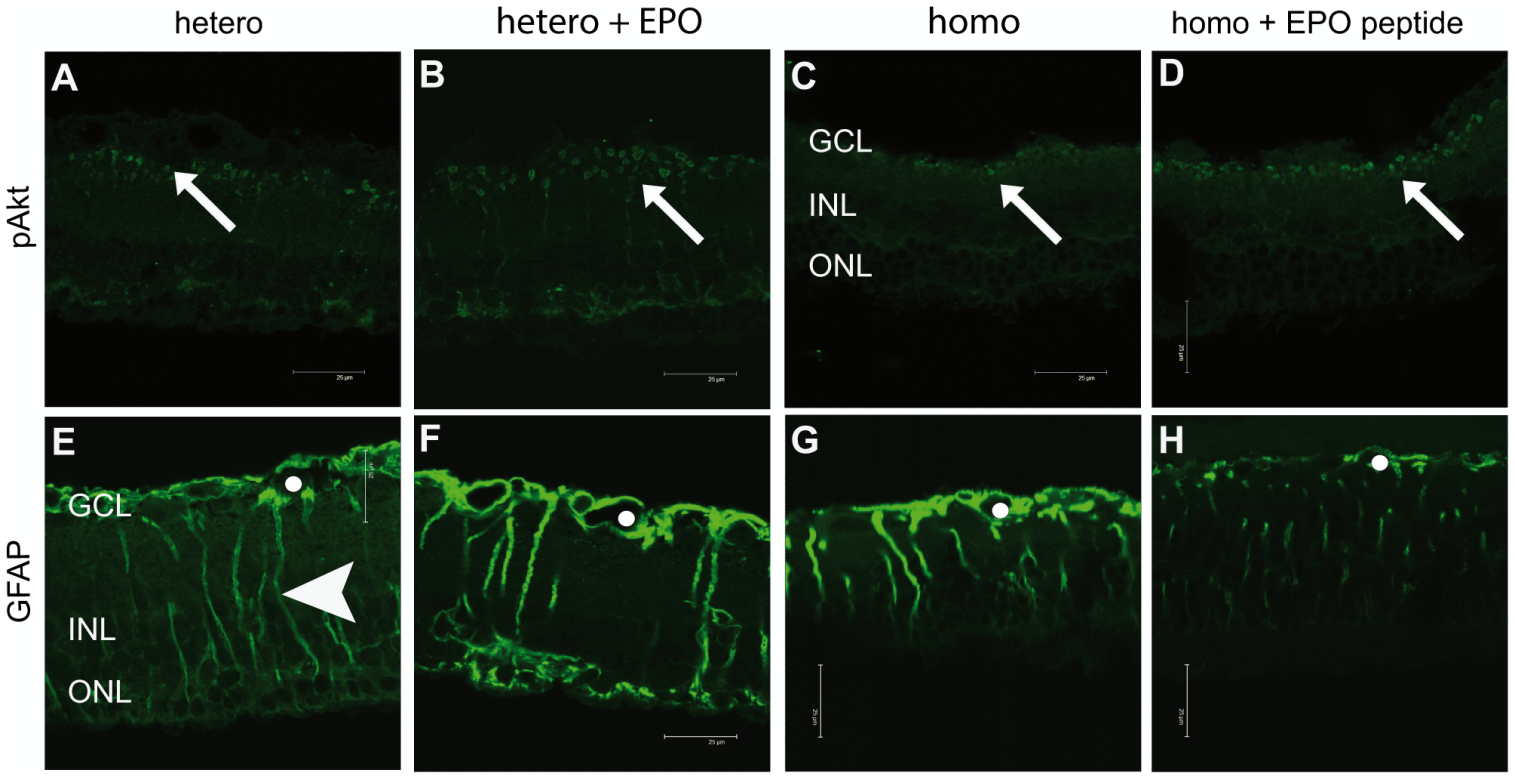
Immunofluorescence staining of pAkt (A–D) and GFAP (E–H) in treated and untreated PKD rats. The scale bar indicates 25 µm. Four week old male PKD rats were treated with 0.5 ml/kg body weight DynEpo three times or EPO-like peptide five days a week intraperitoneally for four weeks. At the age of eight weeks the animals were sacrificed, the eyes enucleated, sections performed and stained for pAkt and GFAP. pAkt (A–D) staining in untreated hetero- (A) and homozygous (C) rats occurred spotlike in the inner plexiforme layer. Upon EPO (B) or EPO-peptide (D) administration, pAkt expression increased markedly, indicating that the protective effect of the treatment is translated via the EPO-receptor. GFAP (E–H), an activation marker for Müller cells, was regulated contrarily. Heterozygous rats (E) express GFAP in a typical Müller cell pattern, surrounding vessels (circle) and with vertical filaments (arrow head). The staining intensity in EPO-treated rats (F) was slightly increased. Homozygous rats (G) express GFAP like heterozygous rats, but with a higher intensity (G). Upon EPO-peptide treatment, GFAP staining decreases markedly in homozygous rats (H). GCL ganglion cell layer, INL inner nuclear layer, ONL outer nuclear layer.

As pAkt is downstream of the EPO-receptor, immunostaining was performed to determine if the protective effect of EPO and EPO-peptide were translated via pAkt. In control heterozygous PKD rats, pAkt staining appeared spot-like in the inner plexiforme layer (figure 8, A arrow). EPO-treated heterozygous PKD rats expressed pAkt much stronger and at more spots (figure 8, B). Homozygous PKD rats did not differ from heterozygous rats (figure 8, C). Upon treatment with EPO-peptide also in homozygous rats the expression of pAkt increased markedly (figure 8, D). The increase in pAkt staining after EPO or EPO-peptide treatment indicates that the protective effect of EPO is transmitted via the EPO-receptor.

### EPO-effect on Müller cell gliosis differs in homo- and heterozygous PKD rats

As the EPO-receptor is, amongst others, expressed in Müller cells, the effect of EPO on GFAP in the PKD model was evaluated by performing immunofluorescence staining for GFAP. GFAP expression differs between homozygous and heterozygous rats. Untreated heterozygous rats express GFAP in a typical Müller cell pattern, surrounding superficial vessels (figure 8, E circle) and vertical filaments (figure 8 arrow head), indicating activation of Müller cells in the PKD model. EPO-treatment did not change this expression pattern (figure 8, F). In homozygous rats the staining pattern was similar to heterozygous rats but with a higher intensity (figure 8, G). As a result of EPO-peptide administration, the staining intensity was reduced (figure 8, H), indicating a reduction in Müller cell activity by EPO-peptide.

## Discussion

In this study we showed the protective effect of EPO and EPO-like peptide on neurons and capillaries in a retinal model of neurodegeneration and secondary vasoregression. The vasoprotective power of EPO was demonstrated by a highly significant reduction in acellular capillaries by 49% (p<0.001) after four weeks of treatment. In the same way EPO-like peptide reduced the formation of acellular capillaries significantly by 63% (p<0.05). Both treatments showed no difference in absolute number of endothelial cells or pericytes. Migrating pericytes decreased in EPO-treated retinae by 23% and upon treatment with EPO-like peptide by 49%, but this reduction did not achieve statistical significance. The unchanged number of endothelial cells and pericytes in contrast to the decrease in acellular capillaries can be attributed to the quantification method. To guarantee the comparison of appropriate retinal areas only fields with at least 30% capillary density were used for quantification. This leads to an increase in endothelial cell and pericytes number by exclusion of highly damaged fields in PKD retinae. One published mechanism by which EPO can mediate its vasoprotective effect is an increase in synthesis of nitric oxide (NO) by endothelial NO-synthase and an increase in VEGF-production [18]–[20]. The vasculoprotective effect of EPO can also be mediated by Tie-1 (Tyrosine kinase with immunoglobulin-like and EGF-like domains 1), Angiopoietin-2 and bFGF (basic fibroblast growth factor) [21].

Besides this vasoprotective effect, we found that intraperitoneal EPO-injection prevents central photoreceptor degeneration. Degeneration of the outer segments was significantly rescued by 26% (p<0.001) and the outer nuclear layer was rescued by 12% (p<0.01). This made a total increase of 11% (p<0.05) in retinal thickness. This effect was not detectable in the peripheral retina. Positive effects of external EPO on neuronal survival and function have been shown in other animal models, e.g. in diabetic retinopathy or autoimmune neuropathy [22], [23]. Given that EPO is also elevated in the retina of diabetic patients in comparison to non diabetics, this might be a compensatory mechanism [24]. Others demonstrated the neuroprotective effect of endogenous EPO in oxygen-induced retinopathy, but could not increase this effect by adding exogenous EPO [25]. One mechanism by which EPO can mediate this neuronal protection is inhibition of retinal macroglial gliosis and a promotion of the production of neuroprotective factors like BDNF and CNTF [26]. Quantification of cell nuclei in the different retinal layers revealed no significant or biologically relevant difference after EPO treatment. This was an unexpected finding because others have shown an effect of EPO on cell size and cell number [27]. The strong proapoptotic impulse in the PKD model may countervail the EPO effect on neuronal number. The missing increase in cell nuclei number, in contrast to the shown increase in layer thickness, indicates that EPO treatment influences cell size and/or extracellular matrix. The amount of extracellular matrix is influenced by matrix metalloproteinases (MMPs). It has been shown that EPO can induce tissue inhibitor of MMPs (TIMP-1), which inhibits MMPs and thereby protects extracellular matrix from degradation. In addition TIMP-1 has antiapoptotic effects [28], [29]. In the PKD model TIMP-1 is 3-fold increased on mRNA level and thereby among the most upregulated 15 genes in this animal model [4]. Exogenous EPO can additionally support this pathophysiological compensation.

For insights in the glial activity and interaction, gene expression of CD74, the invariant chain of MHCII, CNTF (ciliary neurotrophic factor) and bFGF (fibroblast growth factor 2) was analyzed. CD74 significantly decreased by 67% (p<0.01) after EPO-treatment. EPO-like peptide led to a nonsignificant reduction by 36%. Both results indicate a reduction in microglial cell activity. Microglia can interact with Müller cells by secretion of CNTF, leading to an increase in bFGF secretion by Müller cells and thereby a prosurvival signal for photoreceptors [30]. CNTF was not regulated the same way upon EPO or EPO-like peptide administration. EPO decreased CNTF levels by 35% (n.s.). In contrast, EPO-like peptide increased CNTF by 32% (n.s.). Both substances decreased bFGF levels, EPO by 58% (p<0.01) and EPO-like peptide by 29% (n.s.). The decreased bFGF levels were unexpected given the increase in photoreceptor survival.

Immunohistochemistry was performed for pAkt, part of the signaling pathway of EPO-R, and GFAP, a typical gliosis marker. pAkt was localized in the inner plexiforme layer and increased in number of spots and intensity as a result of treatment with EPO or EPO-like peptide. This suggests that the protective effect of this treatment is receptor-mediated. GFAP expression was only affected by EPO-like peptide, showing a apparent decrease in staining intensity.

In conclusion, EPO is a suitable substance to safeguard the neurovascular unit. Its protective effect has been shown in different animal models of neurovascular diseases. Many other authors described positive effects of EPO on the pathomechanism of diabetic retinopathy, e.g. reduced gliosis, increased RPE barrier function or less pericyte loss [26], [31], [32]. Also in Alzheimer's disease, which has a broad overlap with the pathomechanism of the PKD rat, a positive effect of EPO on the neurovascular unit is described [3], [33]–[35].

In summary, we showed the central neuroprotective effect of EPO and its vasoprotective power in a model of retinal neurodegeneration and subsequent vasoregression. Whereas EPO decreased microglial expression of CD74, its effect on macroglia is not distinct. This finding strengthens EPO in its protective capability on the whole neuroglialvascular unit. EPO treatment can be useful in different diseases of the neuroglialvascular unit, e.g. Alzheimer's disease, furthermore treatment with EPO-like peptide, missing the side effects of EPO but still providing its protective function, is possible.

## Supporting Information

Data S1 **The supporting data shows the raw counting of retinal morphology, neurodegeneration and the raw data of the taqman analysis.**
(XLSX)
